# Supplementary material for: Characteristics and actions in high-risk COPD in unstable patients: The EPOCONSUL audit
Source: PLoS One. 2025 Jul 18;20(7):e0327775. doi: 10.1371/journal.pone.0327775 (PMC12273953; doi:10.1371/journal.pone.0327775)
Supplement: S1 Table — (PDF) [file pone.0327775.s001.pdf]

Supplementary Table 1: Caracteristics asociated with oral therapies for COPD

N=287	GesEPOC Phenotype Exacerbator	≥1 hospital admissions for COPD last year	Chronic bronchitis criteria	Post-FEV1<50 % predicted	Peripheral eosinophilia ≥300 mm3	Peripheral eosinophilia ≤100 mm3	Chronic bronchial infection
**Roflumilast**<br>**Not Roflumilast**	51 (86.4)#<br>120 (70.2)	34 (47.2)#<br>63 (29.3)	47 (65.3)#<br>107 (49.8)	51 (70.8)<br>150 (69.8)	7 (14.6)<br>34 (22.4)	13 (27.1)<br>47 (30.9)	24 (33.3)<br>59 (27.4)
**Mucolitics**<br>**Not mucolitics**	69 (75.8)<br>102 (73.4)	44 (39.6)<br>53 (30.1)	72 (64.9)α<br>82 (46.6)	69 (62.2)α<br>132 (75)	10 (11.2)α<br>31 (27.9)	32 (36)<br>28 (25.2)	38 (34.2)<br>45 (25.6)
**Macrolides**<br>**Not macrolides**	84 (74.3)<br>87 (74.4)	47 (33.8)<br>50 (33.8)	78 (56.1)<br>76 (51.4)	104 (74.8)<br>97 (65.5)	23 (25.3)<br>18 (16.5)	18 (19.8)*<br>42 (38.5)	56 (40.2)*<br>27 (18.2)
**Methilxantines**<br>**Not methilxantines**	24 (75)<br>147 (74.2)	10 (22.7)<br>87 (35.8)	20 (45.5)<br>134 (55.1)	36 (81.8)<br>165 (67.9)	11 (30.6)<br>30 (18.3)	11 (30.6)<br>49 (29.9)	6 (13.6)µ<br>77 (31.7)

Note: Chronic bronchial infection: isolates of the same potentially pathogenic microorganism in respiratory samples. Significant differences in roflumilast:# p<0.05; Significant differences in mucolitics:α p<0.05; Significant differences in macrolides:* p<0.05; Significant differences in methilxantines:µ p<0.05
