# Supplementary material for: Characteristics and actions in high-risk COPD in unstable patients: The EPOCONSUL audit
Source: PLoS One. 2025 Jul 18;20(7):e0327775. doi: 10.1371/journal.pone.0327775 (PMC12273953; doi:10.1371/journal.pone.0327775)
Supplement: S2 Table — (PDF) [file pone.0327775.s002.pdf]

Supplementary Table 2: Factors associated with action taken during the visit in high risk level not stability patients

n= 601	**Some action was taken during visit** n= 540 (89.8%)	**No action was taken during visit** n= 61 (10.1%)	**p**
**Clinical Characteristics**			
Gender (male), n (%)	404 (74.8)	38 (62.3)	0.036
Age (years), m (SD)	71 (9.6)	72.6 (8.3)	0.214
Current smokers, n (%)	141 (26.1)	6 (9.8)	0.005
Charlson index ≥3, n, (%)<br>Cardiovascular disease, n (%)<br>Asthma, n (%)	175 (32.4)<br>26 (48.3)<br>78 (18.3)	21 (34.4)<br>23 (37.7)<br>12 (25.5)	0.750<br>0.115<br>0.228
Dyspnea (MRC-m) ≥2, n (%)	451 (83.5)	55 (90.2)	0.177
CAT questionnaire > 10, n (%)	213 (83.9)	29 (85.3)	0.830
Chronic bronchitis criteria, n (%)	286 (53)	22 (36.1)	0.012
Chronic bronchial infection, n (%)	120 (22.2)	12 (19.7)	0.648
Post-FEV_1, % predicted, m (SD)	45.9 (15.5)	42.3 (16)	0.087
BODE value, median, (IQR)	5 (3-6)	6 (3-7)	0.071
≥1 hospital admissions in the last year,n (%)	292 (54.1)	32 (52.5)	0.810
Peripheral eosinophilia, median (IQR)<br>≤100 mm3, n (%)<br>101-299 mm3, n (%)<br>≥ 300 mm3, n (%)	200 (100-300)<br>104 (27.2)<br>191 (49.9)<br>88 (23)	200 (120-300)<br>11 (23.4)<br>25 (53.2)<br>11 (23.4)	0.895<br>0.854
GesEPOC Phenotype Exacerbator	354 (83.3)	39 (84.8)	0.796
LAMA or LABA<br>LAMA-LABA combination<br>LABA+ ICS combination	7 (1.3)<br>128 (23.9)<br>38 87.1)	1 (1.7)<br>6 (10.3)<br>5 (8.6)	0.138

Triple therapy (LAMA+LABA+CSI), n (%)	363 (67.7)	46 (79.3)	
Long-term oxygen therapy, n (%)	226 (41.9)	28 (45.9)	0.544
Home ventilation, n (%)	62 (11.5)	8 (13.1)	0.706
**Care pathway**			
Level of complexity of hospital, n (%)<br>Secondary<br>Tertiary	 116 (21.5)<br>424 (78.5)	 4 (6.6)<br>57 (93.4)	0.006
Public University Hospital, n (%)	404 (74.8)	58 (95.1)	<0.001
Attended in specialized COPD outpatient clinic, n (%)	229 (42.5)	37 (60.7)	0.007
Respiratory care follow-up (years) median, IQR	6.0 (3.8- 9.1)	7.0 (4.4- 10.5)	0.234
Scheduled follow-up visits<br><6 months (ref)<br>≥6 months	 356 (67.7)<br>170 (32.3)	 28 (53.8)<br>24 (46.2)	0.044

Footnote: Data presented as mean (SD) or number (percentage) or median (interquartile range); mMRC: modified Medical Research Council; CAT: COPD Assessment Test; FEV1%: post-bronchodilator FEV1 percent predicted; BODE: body mass index, airflow obstruction, dyspnea, and exercise capacity; GesEPOC: Spanish National Guideline for COPD; Chronic bronchial infection: isolates of the same potentially pathogenic microorganism in respiratory simples; LABA: long-acting beta-2 agonists; LAMA: long-acting antimuscarinic agents; CSI: Inhaled corticosteroids.
