# Supplementary figures and images for: Characteristics and actions in high-risk COPD in unstable patients: The EPOCONSUL audit
Source: PLoS One. 2025 Jul 18;20(7):e0327775. doi: 10.1371/journal.pone.0327775 (PMC12273953; doi:10.1371/journal.pone.0327775)

**Figure 1:** STROBE flow chart of the sampling process

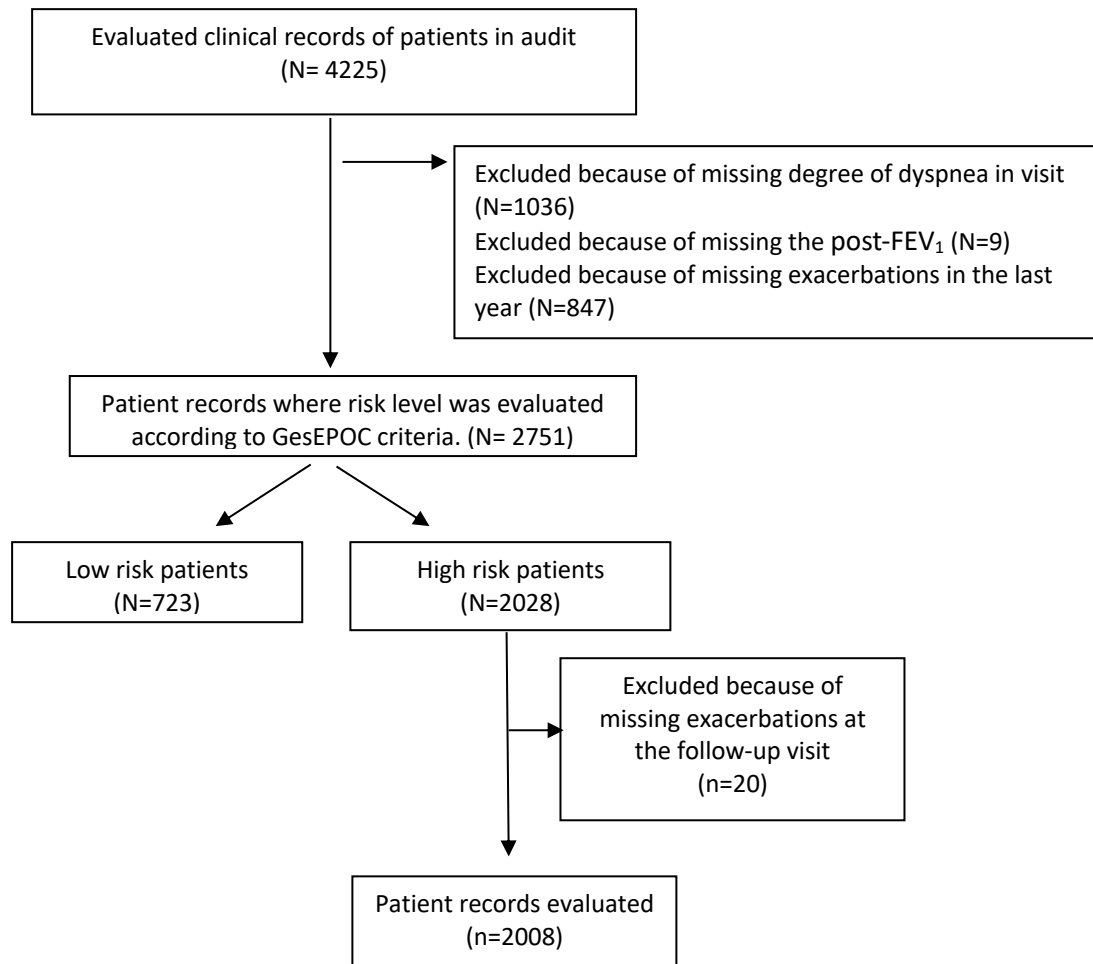

Supplement: S1 Fig — (PDF) [file pone.0327775.s003.pdf]
