# Supplementary material for: Characteristics and actions in high-risk COPD in unstable patients: The EPOCONSUL audit
Source: PLoS One. 2025 Jul 18;20(7):e0327775. doi: 10.1371/journal.pone.0327775 (PMC12273953; doi:10.1371/journal.pone.0327775)
Supplement: S1 Appendix — (PDF) [file pone.0327775.s004.pdf]

APPENDIX

Appendix 1: Participants in 2021 EPOCONSUL study

Andalucía: José Calvo Bonachera. H. Torrecárdenas. Almeria, Virginia Almadana Pacheco. H. U Virgen de la Macarena. Malaga, Francisco Marin Sanchez. H. U. Virgen de la Victoria. Malaga, J. L Lopez Campos. H. U Virgen del Rocio. Sevilla.

Aragón: Maria Angeles Gotor Lazaro. Hospital Universitario Miguel Servet. Ana Boldova. Zaragoza, Hospital Royo Villanova. Zaragoza, Virginia Mo, Cristina Aguilar Paesa. H. Clinico. Zaragoza.

Asturias: Marta Iscar Urrutia, Ana Pando Sandoval, Cristina Hernández González. Hospital Universitario Central de Asturias. Oviedo,

Murcia: Maria Jesús Avilés Inglés. Hospital General. Universitario Reina Sofía, Juan Miguel Sánchez Nieto. Hospital Morales Meseguer, Mª Carmen Fernández Sánchez. H. Universitario Rafael Méndez. Murcia.

Canarias: Marco Acosta Sorense. Hospital Universitario Nuestra Señora de la Candelaria. Tenerife.

Cantabria: Beatriz Abascal. H. U de Valdecilla. Santander.

Castilla y la Mancha: José Alfonso García Guerra. H. Mancha Centro. Alcazar de San Juan. Ciudad Real.

Castilla y León: Ana Pueyo. Hospital Universitario de Burgos, José Luis Fernández Sanchez, Maria Bartol Sanchez, Tamara Clavero Sanchez, Laura Gil Pintor. H. U. de Salamanca.

Cataluña: Miriam Barrecheguren. H Val de Hebron. Barcelona, Noelia Pablos Mateos. H. Sant Joan de Déu de Martorell. Barcelona, Sandra Marin. Hospital Dos de Maig de Barcelona, Annie Navarro. Hospital U Mútua de Terrassa. Barcelona, Elena de Miguel Campos. Hospital Moisés Broggi Sant Joan Despí. Barcelona.

Valencia: Jose Maria Tordera. Hospital Universitario La Fe de Valencia, Dolores Martinez Pitarch. Hospital LLuis Alcanys de Xátiva. Valencia, Marta Palop Cervera. Hospital de Sagunto. Valencia, Lia Alonso Tomás, Marta Solé Delgado. H Arnau de Vilanova. Valencia, Cruz Gonzalez Villaescusa. Hospital Clínico Universitario de Valencia, Eusebi Chiner Vives. H. U. San Juan. Alicante.

Extremadura: Francisca Lourdes Marquez, Luis Miguel Sierra Murillo. Hospital Universitario de Badajoz, Juan Antonio Riesco. H. San Pedro de Alcantara. Mirian Torres González. Hospital Virgen del Puerto de Plasencia,

Baleares: Francisco Fanjul Losa. Hospital Universitario Son Espases. Palma de Mallorca, Antonia Fuster Gomila. Hospital Universitario Son Lltzer. Palma de Mallorca.

Madrid: Soledad Alonso Viteri. H de Torrejon. Madrid, Aurora Solier. H Ramon y Cajal. Madrid, Andrea Yordi. H. Infanta Elena. Valdemoro. Madrid, Nuria Arenas, Blas Rojo.

Hospital Infanta Sofía San Sebastián de los Reyes. Madrid, Juan Luis Rodriguez Hermosa, Gianna Vargas Centanaro. H. Clinico San Carlos. Madrid, Manuel Valle Falcones. H. U Puerta de Hierro Majadahonda. Madrid, Tamara Alonso Perez, Rosa Mar Gómez Punter, Elena García Castillo. H. La Princesa. Madrid, J. De Miguel, Zichen Ji. H. U. Gregorio Marañon. Madrid, Carolina Maria Gotera Rivera. Fundación Jimenez Diaz. Madrid.

Navarra: Jose Espinoza Perez. Complejo Hospitalario de Navarra.

País Vasco: Maria Milagros Iriberri Pascual, Patricia Sobradillo Ecenarro. H de Cruces. Vizcaya, Raquel Sánchez Juez. Hospital Universitario de Basurto. Vizcaya, Cristobal Esteban Gonzalez. Hospital de Galdakao. Vizcaya.
