# Supplementary material for: Characteristics and actions in high-risk COPD in unstable patients: The EPOCONSUL audit
Source: PLoS One. 2025 Jul 18;20(7):e0327775. doi: 10.1371/journal.pone.0327775 (PMC12273953; doi:10.1371/journal.pone.0327775)
Supplement: S2 Appendix — (PDF) [file pone.0327775.s005.pdf]

Appendix 2 The inclusion criteria and exclusion criteria

The inclusion criteria	- patients aged ≥40 years<br>- smokers or ex-smokers (of at least 10 pack-years)<br>- COPD diagnosed on the basis of spirometric tests (FEV1/FVC post-bronchodilation<0.7 or FEV1/FVC pre-bronchodilation<0.7 and FEV1≥80%, if there is no bronchodilation reversibility testing available
The exclusion criteria	- lack of follow-up for at least 1 year in a respiratory outpatient clinic<br>- participating in a clinical trial
