# Supplementary material for: Characteristics and actions in high-risk COPD in unstable patients: The EPOCONSUL audit
Source: PLoS One. 2025 Jul 18;20(7):e0327775. doi: 10.1371/journal.pone.0327775 (PMC12273953; doi:10.1371/journal.pone.0327775)
Supplement: S3 Appendix — (PDF) [file pone.0327775.s006.pdf]

Appendix 3: Risk stratification according to GesEPOC

	LOW RISK (must meet all criteria)	HIGH RISK (must meet at least one criterion)
Obstruction (Post-bronchodilator FEV1%)	≥50%	<50%
Dyspnea (mMRC)	0 – 1	2 - 4
Exacerbations in the last year	0 – 1 exacerbations (without hospitalization)	≥2 exacerbations or ≥1 hospitalization
