# Supplementary material for: Characteristics and actions in high-risk COPD in unstable patients: The EPOCONSUL audit
Source: PLoS One. 2025 Jul 18;20(7):e0327775. doi: 10.1371/journal.pone.0327775 (PMC12273953; doi:10.1371/journal.pone.0327775)
Supplement: Attachment — Submitted filename: Letter of response to Reviewers and Editorial Plos One .docx [file pone.0327775.s008.docx]

Journal: Plos One

Section: Original Research
Type of manuscript: Article

 Revision required [PONE-D-25-12656] - [EMID:c3799199ce51f115]

Title: **Characteristics and actions in high-risk COPD in unstable patients: The EPOCONSUL audit**

Dear Editor Thien Tan Tri Tai Truyen

We would like to thank you. Below are the point-by-point answers to the remarks. You will find all the suggested changes tracked in a marked version of the original manuscript.

The full comments of the Editors are the following:

**Journal Requirements:**

Response:

We are grateful this comment.

Thank you very much. We have verified that the manuscript complies with the style requirements of PLOS ONE.

We have included the following funding statement in the cover letter ‘This study has been promoted and sponsored by the Spanish Society of Pneumology and Thoracic Surgery (SEPAR)]. The funders had no role in the study design, data collection and analysis, decision to publish or preparation of the manuscript’.

Reviewers' comments:

Reviewer's Responses to Questions

**Reviewer Comments:**

**Reviewer #1:**

This study provides valuable real-world insights into high-risk COPD management in Spain, highlighting key gaps in treatment and the importance of specialized care. However, causality cannot be established, and more work is needed to optimize therapy escalation and integrate biomarkers into decision-making.

Following are my comments:

1. Lack of standardized exacerbation criteria

COPD exacerbations are self-reported at follow-up visits, which introduces recall bias.

Future studies could use objective biomarkers (e.g., inflammatory markers, spirometry trends) to better define exacerbation severity.

**RESPONSE:** We welcome these comments, and include in the discussion this comment:

“The correct assessment of COPD exacerbations will be crucial to assess risk, the degree of clinical control in COPD and to plan therapeutic interventions. Currently the identification of exacerbating patients is based on clinical records and/or patient recall^24^. However, these are not always reported. Thus, several studies suggest that about half of exacerbations are not reported, although they are associated with a worsening quality of life and an increased risk of subsequent hospitalisation^2526^. On the other hand, it should be remembered that definitions of COPD exacerbations are mainly based on clinical presentation, which can be non-specific and lead to misdiagnosis. The use of biomarkers could provide a more objective and specific definition of COPD exacerbations and allow for a more accurate assessment of progression or response to treatment. Future research is needed to establish a better definition of COPD exacerbations through the use of biomarkers to improve diagnostic accuracy, more personalised treatment and potentially improve clinical outcomes and reduce healthcare costs.”

2. Potential Confounding in Treatment Patterns

The study finds that oral therapies (macrolides, mucolytics, roflumilast) are used more often in unstable patients.

However, prescription patterns may reflect disease severity rather than therapeutic effectiveness.

A propensity-matched analysis could help determine whether treatment choices truly influence exacerbation risk.

**RESPONSE:** We welcome these comments, which we include in the discussion :

“In our analysis, being a cross-sectional observational study, prescription patterns may reflect disease severity rather than therapeutic effectiveness. Furthermore, it should be borne in mind that the clinical presentation of COPD is highly variable, and that other factors associated with lack of clinical control and stability include associated comorbidities, adherence, among others.”

3. Therapeutic Inertia: Lack of Detailed Exploration

More than 50% of unstable patients did not receive treatment changes, yet the reasons for this are not explored in depth.

Potential factors include: Physician hesitation due to comorbidities, Patient adherence issues, Lack of clear guidelines for therapy escalation.

Qualitative interviews with physicians could provide insights into clinical decision-making.

**RESPONSE** We appreciate these considerations in relation to non-performance at the visit. Our study is a retrospective audit of clinical records, where conditioning factors such as knowledge, experience of the professional attending the consultation, availability of time or means, or clinical considerations of the patient could not be evaluated.

We include a commentary in the discussion as a line of interest for further study that may help to identify limitations in the implementation of good clinical practice guidelines and conditioning factors in clinical practice.

“Little is known about the reasons for the lack of action when treatment goals are not met. Studies have shown that physicians are willing to take actions when they deem them appropriate, but there is a disconnection between their perception and the actual degree of control of COPD in uncontrolled patients, suggesting that physicians do not feel the need to switch up therapies or increase doses if the patient is showing positive signs of improvement or maintenance^34^. Studies are needed to analyze the reasons for the non-implementation of clinical practice guidelines and the conditioning factors in clinical practice.”

4. Need for Biomarker Integration

The study discusses peripheral eosinophilia as a potential marker for Th2 inflammation and treatment selection.

However, it does not analyze how eosinophil-guided therapy affected stability outcomes.

Future research should explore biomarker-driven treatment personalization.

**RESPONSE: W**e appreciate these considerations. We include a commentary in the discussion as a line of interest for further study

““The use of specific biomarkers in the future could allow for a better diagnostic approach in characterising exacerbations but could also offer a more personalised treatment and potentially improve clinical outcomes.”

6. Future Research Directions:

A. Longitudinal Studies

Follow-up studies should track exacerbation rates and lung function over time to assess the long-term impact of different treatment strategies.

B. Machine Learning for Risk Prediction

AI-based models could predict which high-risk patients are likely to deteriorate and personalize therapy accordingly.

C. Biomarker-Driven Treatment

Integrating blood eosinophils, sputum biomarkers, and genetics could help tailor COPD treatments more effectively.

**RESPONSE:** We appreciate these considerations. We include future directions

“Artificial intelligence-based models can significantly enhance the prediction of deterioration in high-risk COPD patients and personalize therapy. AI algorithms, including machine learning and deep learning, can analyze diverse data types such as clinical records, patient-reported outcomes, environmental factors, and genetic information to predict exacerbations and disease progression. Models using support vector machines, boosting, and deep neural networks have shown promise in predicting COPD exacerbations with high accuracy ^39^”

Reviewer #2: It is a good study helping us understand the characteristics of high risk COPD patient and will helpful in taking the needed intervention for better management. It will helps in identifying the patients at risk for exacerbations and in turn will guide us in a better way to decrease the morbidity and mortility .

RESPONSE: We sincerely thank you for your positive feedback and encouraging comments. We greatly appreciate your review and support of our work.

Reviewer #3: The study tackles persistent instability in high-risk patients, a clinical issue that is extremely pertinent to the therapy of COPD. One of its main advantages is the use of a national dataset in the real-world audit technique, which greatly expands on the body of current literature.
As the study is very essential to the investigation of COPD clearer figures ,Figures 1-4 visual segmentation and legends are unclear.

RESPONSE: We sincerely thank you for your positive feedback and encouraging comments. We greatly appreciate your review and support of our work.

We have clarified the data in the figures 1A, 1B and 1C
