# Supplementary material for: Characteristics and actions in high-risk COPD in unstable patients: The EPOCONSUL audit
Source: PLoS One. 2025 Jul 18;20(7):e0327775. doi: 10.1371/journal.pone.0327775 (PMC12273953; doi:10.1371/journal.pone.0327775)
Supplement: Attachment — Submitted filename: Response to Reviewers.docx [file pone.0327775.s009.docx]

Journal: Plos One

Section: Original Research
Type of manuscript: Article

 Revision required [PONE-D-25-12656] - [EMID:c3799199ce51f115]

Title: Characteristics and actions in high-risk COPD in unstable patients: The EPOCONSUL audit

Dear Editor Thien Tan Tri Tai Truyen

We would like to thank you. Below are the point-by-point answers to the remarks. You will find all the suggested changes tracked in a marked version of the original manuscript.

Reviewers' comments:

Reviewer's Responses to Questions

**Reviewer #3:**

6. Review Comments to the Author
Reviewer #3: Recall bias is introduced by the operational definition of "non-stability," which is predicated on patient-reported exacerbations within three months.

**RESPONSE:** Taking into account your recommendation, we have clarified the definition of “no stability”, including this additional information

In abstract:” Objective: to evaluate the clinical characteristics of high-risk COPD patients considered unstable for having had moderate or severe exacerbations of COPD in the three months prior to the audited review visit based on information extracted from the medical record documenting health interactions prior to the visit.”

In the methodology section:

“Non-stability (non-stable patient) was defined on the basis of having moderate or severe exacerbations of COPD in the three months prior to the audited review visit. All data were verified by clinical investigators with access to the original documentation. Data were systematically extracted from medical records documenting health interactions prior to the audited visit to ensure completeness and consistency with records made at the audited visit.”

Reviewer #3: 
The finding that 56% of unstable patients had no change in therapy warrants deeper analysis.

**RESPONSE:** Taking into account your recommendation, we have include in results table 5: Factors associated with no change in therapy during the visit in high risk level not stability patients

We have and include in the discussion this comment: “In our analysis, few clinical characteristics were associated with the dependent variable no change in COPD treatment at the visit in a patient with high-risk COPD who reported exacerbations at the visit. In the multivariate logistic regression model, only being treated in a university hospital (OR 3.08, 95% CI 1.71-5.53; p<0.001) was found as an associated factor, being less likely not to make changes in the active smoker (OR 0.63, 95% CI 0.44-0.90; p=0.011), and the presence of chronic bronchial infection (OR 0.50, 95% CI 0.33-0.76; p=0.001).”

Reviewer #3: Although important clinical variables are adjusted for in multivariate models, it is uncertain whether interaction effects.

**RESPONSE:** Taking into account your recommendation. This aspect is included as a limitation.

“the evaluation of the possible interaction effect of more than two variables was not evaluated because it was difficult to interpret from a clinical point of view, given the limitations of being a retrospective study with unavailable data..”

we have not evaluated the interaction from a statistical point of view because, being a retrospective study whose source is the medical records, the evaluation of the possible interaction effect of more than two variables would be difficult to interpret from a clinical point of view.

Reviewer #3: The discussion highlights the importance of Th2 inflammation and eosinophils, but it does not completely incorporate their significance into the findings or clinical implications.

**RESPONSE:** Taking into account your recommendation, we have and include in the discussion this comment:

“In our population of unstable high-risk patients, 85% had a frequent exacerbator phenotype and 67% were on maintenance treatment with triple therapy. Of these, 23% had peripheral blood eosinophil counts ≥300 cells/μL, being a priori a population in which treatment optimization should be considered, assessing escalation to triple therapy and/or could benefit from biologic therapy. Evidence suggests that 20-40% of COPD patients have Th2 airway inflammation as measured by sputum eosinophilia [37,38]. This endotype is associated with increased risk of exacerbations. Elevated blood eosinophil counts (BEC), typically ≥300 cells/μL, serve as a practical biomarker, identify patients more likely to benefit from emerging biologics. Recent trials demonstrate that targeting T2 pathways produces significant reductions in exacerbations and improves lung function in COPD patients who persist with exacerbations despite optimization of inhaled therapy [38], reflecting a move toward precision medicine in this heterogeneous disease. In the near future, artificial intelligence-based models may help to identify this group of patients earlier in order to provide more personalized medicine for high-risk COPD patients. AI algorithms, including machine learning and deep learning, can analyze various types of data, such as medical records, patient-reported outcomes, environmental factors and genetic information, to predict exacerbations and disease progression.”
